# Histamine H2 receptor antagonist exhibited comparable all-cause mortality-decreasing effect as β-blockers in critically ill patients with heart failure: a cohort study

**DOI:** 10.3389/fphar.2023.1273640

**Published:** 2023-11-13

**Authors:** Xue-Sha Zhang, Wen-Ke Cai, Ping Wang, Ran Xu, Sun-Jun Yin, Yan-Hua Huang, Yu Guo, Fang-Fang Jiang, Jian-Mei Pan, Yi-Hua Li, Gong-Hao He

**Affiliations:** ^1^ Department of Clinical Pharmacy, 920th Hospital of Joint Logistics Support Force, Kunming, China; ^2^ College of Pharmacy, Dali University, Dali, China; ^3^ Department of Cardiothoracic Surgery, 920th Hospital of Joint Logistics Support Force, Kunming, China

**Keywords:** histamine H2 receptor antagonists, β-blockers, heart failure, mortality, medical information mart for intensive care

## Abstract

**Background:** Our previous study reported that histamine H2 receptor antagonists (H2RAs) exposure was associated with decreased mortality in critically ill patients with heart failure (HF) through the same pharmacological mechanism as β-blockers. However, population-based clinical study directly comparing the efficacy of H2RAs and β-blockers on mortality of HF patients are still lacking. This study aims to compare the association difference of H2RAs and β-blockers on mortality in critically ill patients with HF using the Medical Information Mart for Intensive Care III database (MIMIC-III).

**Methods:** Study population was divided into 4 groups: β-blockers + H2RAs group, β-blockers group, H2RAs group, and Non-β-blockers + Non-H2RAs group. Kaplan–Meier curves and multivariable Cox regression models were employed to evaluate the differences of all-cause mortalities among the 4 groups. Propensity score matching (PSM) was used to increase comparability of four groups.

**Results:** A total of 5593 patients were included. After PSM, multivariate analyses showed that patients in H2RAs group had close all-cause mortality with patients in β-blockers group. Furthermore, 30-day, 1-year, 5-year and 10-year all-mortality of patients in β-blockers + H2RAs group were significantly lower than those of patients in β-blockers group, respectively (HR: 0.64, 95%CI: 0.50–0.82 for 30-day; HR: 0.80, 95%CI: 0.69–0.93 for 1-year mortality; HR: 0.83, 95%CI: 0.74–0.93 for 5-year mortality; and HR: 0.85, 95%CI: 0.76–0.94 for 10-year mortality, respectively).

**Conclusion:** H2RAs exposure exhibited comparable all-cause mortality-decreasing effect as β-blockers; and, furthermore, H2RAs and β-blockers had additive or synergistic interactions to improve survival in critically ill patients with HF.

## Introduction

With acceleration of world population aging currently, heart failure (HF) becomes a major global health burden and causes severe patient harm as well as high healthcare costs (Baman and Ahmad, 2020; Heidenreich et al., 2022). However, despite improvements in treatment of this disease, considerable first-line anti-HF drugs recommended by the present popular guidelines still do not exhibit substantial benefits in increasing survival of different kinds of HF patients (Kotecha et al., 2014; Fudim et al., 2018; Heidenreich et al., 2022). Therefore, new drug targets and corresponding novel therapies that are able to improve survival rate of HF patients, especially for those in critically ill situation, unfortunately remain an important unmet medical need yet.

For several decades, it has long been suggested that histamine H2 receptor (H2R) was highly related to occurrence and development of HF and, as a result, might be a novel promising anti-HF target (Hara et al., 2002; Leary et al., 2014; Leary et al., 2016; Saheera et al., 2022). In this regard, the anti-HF effects of histamine H2 receptor antagonists (H2RAs) consequently gained particular attention in the following related clinical investigations, based on which it was suggested that H2RAs had beneficial effects on short- and medium-term outcomes of HF patients (Adelborg et al., 2018; Zhang et al., 2018; Huang et al., 2022) and exhibited relatively safe profile in cardiovascular system (Meng et al., 2023). Nevertheless, these findings also raised another key question that whether the use of H2RAs has considerable treatment value as compared with other traditional anti-HF drugs, elucidating of which will eventually provide further evidence to promote the clinical application of H2RAs in treatment of HF.

As one of the first-line anti-HF drugs, β-blockers are well acknowledged to be a cornerstone in treatment of chronic HF patients, especially for those with reduced ejection fraction (HFrEF) (Cleland et al., 2018; Baman and Ahmad, 2020). But relatively serious adverse effects and medical contraindications of these drugs, such as gastrointestinal irritation, central nervous system symptoms and bronchial asthma, not only limit their widespread use in different kinds of HF patients but also pose serious problems regarding their safety. Therefore, alternative drugs with certain potential substitutional properties to replace β-blockers are of special interest for both clinicians and HF patients particularly with β-blocker intolerance. Luckily, findings of previous investigations made H2RAs an ideal possible option for specific HF patients bearing in mind that H2R is pharmacologically similar with β1 receptor in activating stimulatory G-proteins in myocardium (Bristow et al., 1982; Del Valle and Gantz, 1997). It has been reported that improvement in cardiac function induced by certain H2RA and β-blocker was parallel (Potnuri et al., 2018) and these 2 kinds of drugs had additive effects on cardiac performance in dogs with pacing-induced HF (Takahama et al., 2010). However, population-based clinical studies directly comparing the efficacy of H2RA and β-blocker in treatment of HF are still lacking.

Based on the above background, in order to further evaluate the treatment value of H2RAs among HF patients, we conducted so far the first large retrospective study based on the open-source Medical Information Mart for Intensive Care III database version 1.4 (MIMIC-III v1.4) to compare the effects of H2RAs *versus* β-blockers on short- and long-term mortalities of HF patients admitted to ICU, hoping to provide novel population-based evidence regarding the clinical value of H2RAs in anti-HF treatment.

## Methods

### Data source

The cohort data were obtained from the freely available MIMIC-III v1.4 database. It contains information of more than 40,000 patients admitted to critical care units of Beth Israel Deaconess Medical Center (Boston, Massachusetts, United States) between 2001 and 2012 (Johnson et al., 2016). Our study conformed to Reporting of Studies Conducted using Observational Routinely Collected Data for Pharmacoepidemiology (RECORD-PE) reporting guidelines and obtained access to the data extraction by completing the Protecting Human Research Participant exam (certification number: 50081003 and 38884075) (Langan et al., 2018). Institutional Review Board (IRB) informed consent and approval were not required for the database since the information related to patient privacy has been protected.

### Study population and groups

The diagnosis of HF was according to the International Classification of Diseases 9th Edition (ICD-9) code (Supplementary Table S1). The inclusions criteria of this study were: 1) adult patients with HF diagnosis within the top 3 of their total diagnoses, 2) patients admitted to the Intensive Care Unit (ICU). For patients exposed to H2RAs or β-blockers with multiple admissions, the first admission during which they exposed to either of these 2 kinds of drugs was selected. For patients not exposed to H2RAs or β-blockers with multiple admissions, the first admission was selected. The exclusions criteria of this study were: 1) patients with age < 18, 2) patients with wrong information, 3) patients exposed to both H2RAs and β-blockers but were not exposed during the same admission.

According to H2RAs and β-blockers exposure status, the present study comprised 4 population groups: β-blockers + H2RAs group, β-blockers group, H2RAs group, and Non-β-blockers + Non-H2RAs group. The β-blockers + H2RAs group included patients exposed to both H2RAs and β-blockers during the same admission. The β-blockers group included patients only exposed to β-blockers during admission. The H2RAs group included patients only exposed to H2RAs. The Non-β-blockers + Non-H2RAs group included patients exposed to neither H2RAs nor β-blockers.

### Data extraction

Baseline characteristics, such as physical characteristics, vital signs, laboratory parameters, co-morbidities, and medications, were extracted from MIMIC-III database by Structured Query Language. Physical characteristics included age, gender, height, weight, religion and language. Vital signs were heart rate (HR), diastolic blood pressure (DBP), systolic blood pressure (SBP), oxygen saturation, and respiratory rate (RR). Laboratory parameters were red blood cell count (RBC), white blood cell count (WBC), platelet count, glucose, blood sodium, blood magnesium, blood calcium, blood urea nitrogen (BUN), and urine output. Co-morbidities included atrial fibrillation, myocardial infarction, coronary atherosclerosis, hypertension, venous thrombosis, anemia, pneumonia, diabetes, duodenal ulcer, gastritis, gastric ulcer, gastrointestinal bleeding, acute kidney failure, and septic shock. Medications included renin angiotensin aldosterone system (RAAS) inhibitors, diuretics, inotropic agents, adrenaline receptor antagonist, calcium channel blockers (CCB), proton pump inhibitors (PPIs), anticoagulants, and antiplatelet drugs. In addition, we collected other information such as length of stay (LOS), sequential organ failure assessment (SOFA), simplified acute physiology score III (SAPS III), left ventricular ejection fraction (LVEF), use of ventilator, and continuous renal replacement therapy (CRRT). The first measurement of the continuous variable was selected during admission. The missing data of all variables were less than 15% (Supplementary Table S2).

## Primary and secondary outcomes

The primary outcomes of the present study were 30-day, 90-day, 1-year, 5-year, and 10-year all-cause mortalities. All-cause mortality at different time periods were defined as death observed within these time periods of admission. The date of out-of-hospital death was extracted from the Social Security Death Index records. The secondary outcomes include hospital LOS, ICU LOS, hospital mortality, and ICU mortality. The hospital LOS were calculated from the date of admission and discharge and the ICU LOS was directly extracted from the database. Hospital and ICU mortality was defined as death that occurred during admission and admission to the ICU, respectively.

### Statistical analysis

Baseline characteristics were presented as mean ± standard deviation (SD) for continuous variables with normal distribution. For continuous variables with non-normal distribution, they were summarized as median and interquartile range (IQR). Mean differences between multiple groups were compared by one-way analysis of variance and medians were tested by the Kruskal–Wallis test. Categorical variables were summarized by number and percentages and assessed by χ2 test. Propensity score matching (PSM) was used to minimize selection bias and increase comparability between groups (Zhang, 2017). According to the population size of different groups, patients in β-blockers + H2RAs group were matched (1:1) to corresponding patients in β-blockers group and patients in β-blockers group were also matched (1:1) to corresponding patients in Non-β-blockers + Non-H2RAs group. However, patients in H2RAs group were matched (1:4) to corresponding patients in β-blockers group. A variable can be considered as a balance between groups when the matched variable’s standardized mean difference (SMD) < 0.1 (Zhang, 2017).

The Kaplan-Meier curve and log-rank test were applied to calculate the cumulative mortality of 30-day, 90-day, 1-year, 5-year, and 10-year among the 4 groups. The Kaplan-Meier curves before and after PSM were plotted respectively. In order to adjust the effect of confounding variables such as physical characteristics, laboratory parameters, co-morbidities, and medications, the multivariate Cox regression model was created to compare the all-cause mortality of each group at different time periods and those results were presented by forest plots. Additionally, subgroup analyses stratified by gender were further performed. When comparing 2 groups, a *p*-value < 0.05 was considered to be significant. For pairwise comparisons among the 4 groups, *p*-value was calculated by Bonferroni correction (Armstrong, 2014). SPSS (version 18.0, IBM Corp, Armonk, NY, United States) and R 3.5.3 software for windows were used for all statistical analyses.

## Results

### Baseline characteristics of the study population

**FIGURE 1:**
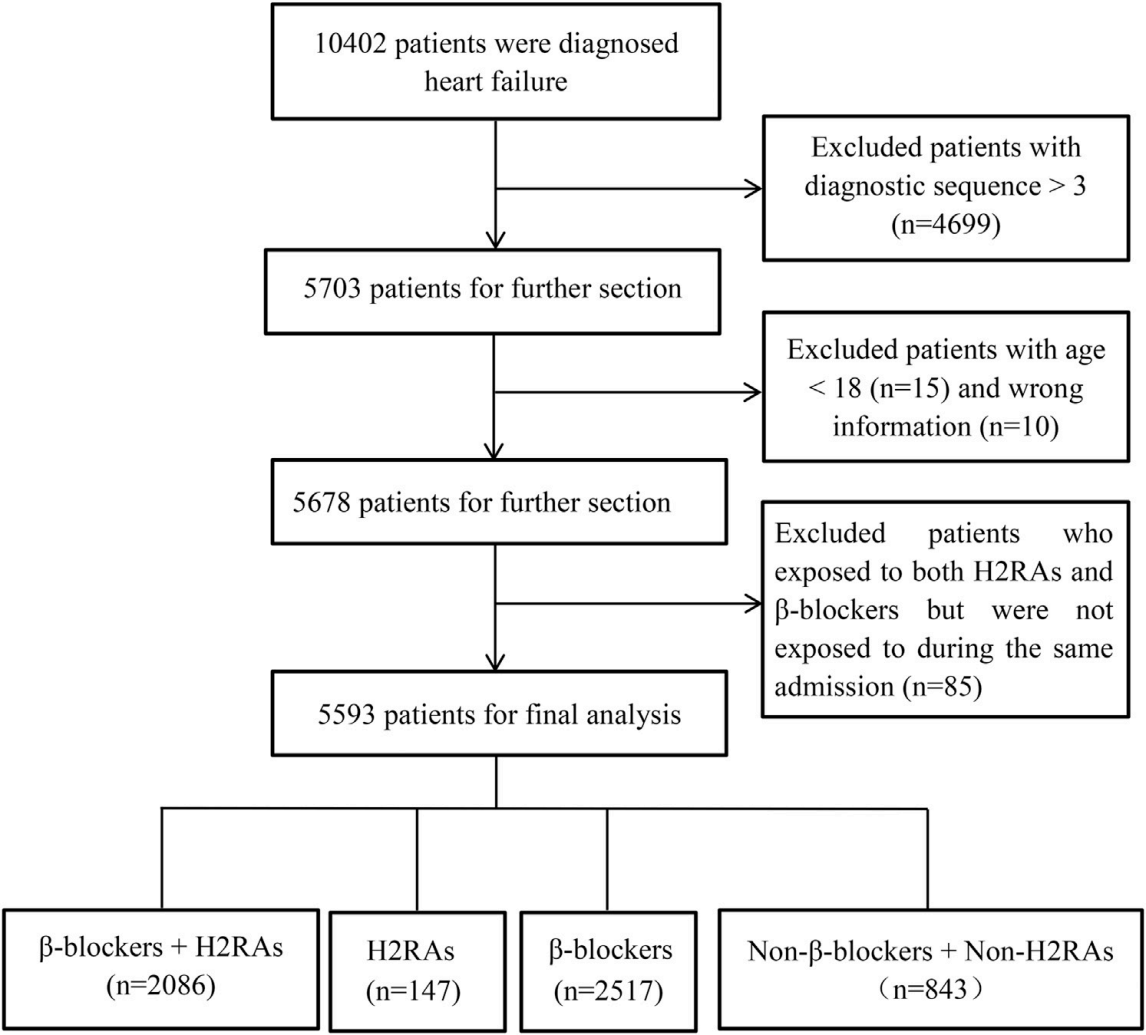
Selection of study population from MIMIC-Ⅲ database.

A total of 10,402 patients were diagnosed with HF in the database. Among them, HF diagnosis of 4699 patients fell out of their respective top 3 diagnoses. Besides, 15 patients were younger than 18 years old, 10 patients had missing information, and 85 patients were exposed to both H2RAs and β-blockers but were not exposed during the same admission. After excluding these patients, 5593 patients were finally included in the present study, containing 2086 patients in β-blockers + H2RAs group, 2517 patients in β-blockers group, 147 patients in H2RAs group and 843 patients in Non-β-blockers + Non-H2RAs group (Figure 1).

The baseline characteristics of the 4 groups were summarized in Table 1, which showed statistically significant differences for age, SOFA, LVEF, coronary atherosclerosis diagnosis, hypertension diagnosis, diuretics exposure, CCB exposure and RAAS inhibitors exposure (*p* < 0.05, respectively) among these groups. However, among the 4 groups, BMI, SAPSⅢ, HR, blood sodium, urine output, gastric ulcer diagnosis, gastritis diagnosis, septic shock diagnosis, and adrenaline receptor antagonist exposure were comparable (*p* > 0.05).

**TABLE 1:** Baseline characteristics in the four groups before matching.

	β-blockers + H2RAs (n = 2086)	β-blockers (n = 2517)	H2RAs (n = 147)	Non-β-blockers + Non-H2RAs (n = 843)	*p*-value	SMD
Age, years	71.04 ± 12.95	74.32 ± 13.29	71.13 ± 15.43	73.14 ± 13.88	<0.001	0.143
Gender, female, n (%)	913 (43.8)	1197 (47.6)	85 (57.8)	412 (48.9)	0.001	0.146
BMI, kg/m^2^	28.86 ± 6.70	28.71 ± 6.77	28.76 ± 7.56	28.79 ± 7.15	0.912	0.011
SOFA	4.78 ± 2.76	3.86 ± 2.51	4.84 ± 3.26	4.26 ± 3.02	<0.001	0.202
SAPSⅢ	43.97 ± 17.79	45.20 ± 17.13	45.21 ± 21.42	45.20 ± 21.29	0.115	0.033
CRRT, n (%)	59 (2.8)	42 (1.7)	5 (3.4)	19 (2.3)	0.047	0.062
Use of ventilator, n (%)	1363 (65.3)	706 (28.0)	83 (56.5)	331 (39.3)	<0.001	0.453
Language, English, n (%)	887 (42.5)	1131 (44.9)	58 (39.5)	638 (75.7)	<0.001	0.398
Religion, Catholic, n (%)	858 (41.1)	926 (36.8)	55 (37.4)	307 (36.4)	<0.001	0.259
Vital signs						
HR	82.20 ± 18.46	81.63 ± 0.95	81.97 ± 20.46	83.40 ± 19.09	0.165	0.047
SBP, mmHg	120.67 ± 23.44	124.82 ± 24.90	120.37 ± 22.37	120.39 ± 23.27	<0.001	0.095
DBP, mmHg	61.65 ± 14.95	64.24 ± 16.91	61.51 ± 15.07	59.19 ± 15.11	<0.001	0.163
Oxygen saturation, (%)	97.88 ± 3.11	96.78 ± 3.48	97.20 ± 3.34	96.75 ± 3.47	<0.001	0.191
RR	17.01 ± 5.70	19.98 ± 5.61	18.80 ± 6.24	19.09 ± 6.31	<0.001	0.261
Laboratory parameters						
RBC, m/μL	3.9 (3.4–4.4)	3.9 (3.4–4.4)	4.0 (3.5–4.5)	3.8 (3.3–4.3)	0.003	0.139
WBC, k/μL	9.4 (7.0–12.8)	9.7 (7.3–13.2)	10.2 (7.4–13.4)	10.5 (7.6–14.0)	<0.001	0.111
Platelet count, k/μL	220 (170–278)	233 (183–300)	244 (170–308)	229 (171–293)	<0.001	0.095
Glucose, mg/dL	126 (105–165)	135 (109–182)	127 (97–175)	132 (106–170)	<0.001	0.085
Blood sodium, mEq/L	139 (136–141)	138 (136–141)	139 (136–141)	139 (136–141)	0.331	0.036
Blood magnesium, mg/dL	2.0 (1.8–2.3)	2.0 (1.8–2.2)	2.0 (1.8–2.3)	2.0 (1.8–2.2)	<0.001	0.110
Blood calcium, mg/dL	8.7 (8.3–9.2)	8.7 (8.3–9.1)	8.8 (8.3–9.1)	8.6 (8.1–9.0)	<0.001	0.135
BUN, mg/dL	23 (17–34)	28 (19–45)	21 (16–35)	28 (18–44)	<0.001	0.236
Urine output, L	1.6 (1.0–2.3)	1.8 (1.0–2.6)	1.6 (1.0–2.3)	1.8 (1.0–2.6)	0.231	0.083
LVEF, n (%)					<0.001	0.211
10%–35%	655 (31.4)	888 (35.3)	35 (23.8)	256 (30.4)		
35%–55%	1163 (55.8)	1270 (50.5)	81 (55.1)	433 (51.4)		
55%–70%	212 (10.2)	266 (10.6)	18 (12.2)	121 (14.4)		
>70%	56 (2.7)	93 (3.7)	13 (8.8)	33 (3.9)		
Co-morbidities, n (%)						
Atrial fibrillation	1006 (48.2)	1127 (44.8)	58 (39.5)	299 (35.5)	<0.001	0.148
Myocardial infarction	391 (18.7)	509 (20.2)	21 (14.3)	115 (13.6)	<0.001	0.108
Coronary atherosclerosis	1181 (56.6)	1149 (45.6)	45 (30.6)	299 (35.5)	<0.001	0.304
Hypertension	995 (47.7)	1028 (40.8)	61 (41.5)	300 (35.6)	<0.001	0.126
Venous thrombosis	110 (5.3)	119 (4.7)	6 (4.1)	17 (2.0)	0.002	0.093
Anemia	585 (28.0)	863 (34.3)	41 (27.9)	221 (26.2)	<0.001	0.089
Pneumonia	270 (12.9)	454 (18.0)	33 (22.4)	154 (18.3)	<0.001	0.127
Diabetes	804 (38.5)	969 (38.5)	52 (35.4)	281 (33.3)	0.037	0.065
Duodenal ulcer	3 (0.1)	21 (0.8)	0 (0.0)	9 (1.1)	0.003	0.095
Gastric ulcer	8 (0.4)	22 (0.9)	0 (0.0)	8 (0.9)	0.110	0.083
Gastrointestinal bleeding	47 (2.3)	188 (7.5)	1 (0.7)	66 (7.8)	<0.001	0.226
Gastritis	28 (1.3)	54 (2.1)	0 (0.0)	14 (1.7)	0.070	0.114
Acute kidney failure	533 (25.6)	927 (36.8)	32 (21.8)	217 (25.7)	<0.001	0.168
Septic shock	27 (1.3)	43 (1.7)	5 (3.4)	14 (1.7)	0.213	0.071
Medications, n (%)						
RAAS inhibitors	1316 (63.1)	1575 (62.6)	54 (36.7)	131 (15.5)	<0.001	0.634
Diuretics	1936 (92.8)	2166 (86.1)	130 (88.4)	289 (34.3)	<0.001	0.760
Inotropic agents	1129 (54.1)	963 (38.3)	73 (49.7)	318 (37.7)	<0.001	0.205
Adrenaline receptor antagonist	8 (0.4)	6 (0.2)	0 (0.0)	0 (0.0)	0.264	0.057
CCB	644 (30.9)	816 (32.4)	30 (20.4)	96 (11.4)	<0.001	0.303
PPIs	1079 (51.7)	1887 (75.0)	56 (38.1)	287 (34.0)	<0.001	0.487
Anticoagulants	1776 (85.1)	2263 (89.9)	122 (83.0)	498 (59.1)	<0.001	0.386
Antiplatelet drugs	1825 (87.5)	1963 (78.0)	95 (64.6)	193 (22.9)	<0.001	0.844

Abbreviations: H2RA, histamine H2 receptor antagonist; SMD, standardized mean difference; BMI, body mass index; SOFA, sequential organ failure assessment score; SAPSⅢ, simplified acute physiology score Ⅲ; CRRT, Continuous renal replacement therapy; HR, heart rate; SBP, systolic blood pressure; DBP, diastolic blood pressure; RR, respiratory rate; WBC, white blood cell; RBC, red blood cell; BUN, blood urea nitrogen; LVEF, left ventricular ejection fraction; RAAS, renin angiotensin aldosterone system; CCB, calcium channel blockers; PPIs, proton pump inhibitors; ICU, indicates intensive care unit; LOS, length of stay

## Associations between H2RAs/β-blockers and clinical outcomes of critically ill patients with HF

**FIGURE 2:**
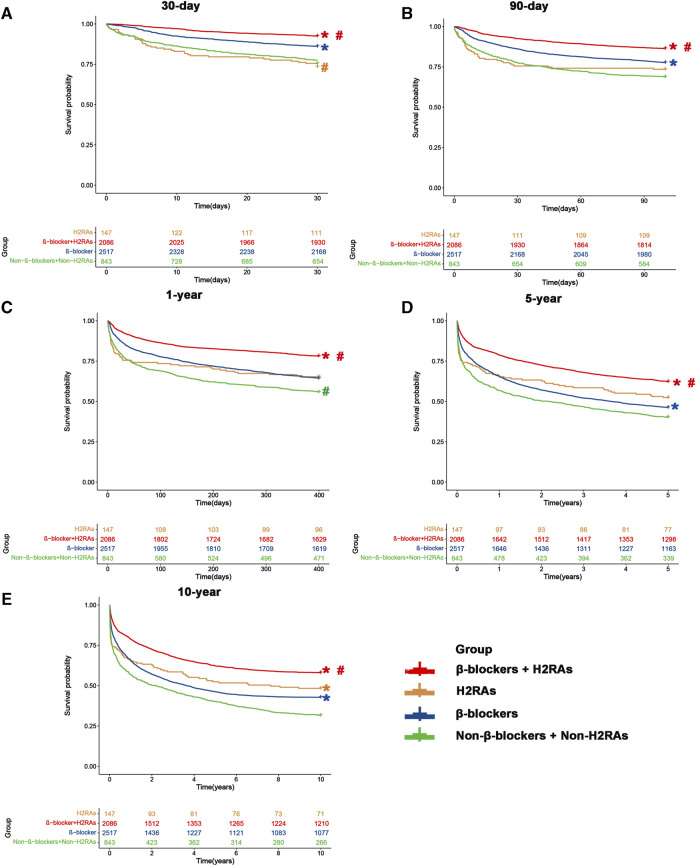
Kaplan-Meier survival curves of four groups before matching. **(A)** 30-day mortality; **(B)** 90-day mortality; **(C)** 1-year mortality; **(D)** 5-year mortality; **(E)** 10-year mortality. #*p* < 0.0083 (0.05/6) *versus* β-blockers group after Bonferroni correction. *p < 0.0083 (0.05/6) *versus* Non-β-blockers + Non-H2RAs group after Bonferroni correction.

We first evaluated the differences of all-cause mortalities among the 4 groups by Kaplan-Meier curves (Figure 2), which showed that 30-day, 90-day, 1-year, 5-year, and 10-year all-cause mortality of patients in β-blockers group were significantly lower than those of Non-β-blockers + Non-H2RAs group at Bonferroni correction level (*p* < 0.05/6 = 0.0083), respectively. Furthermore, although 30-day all-cause mortality of patients in H2RAs group was higher than that of patients in β-blockers group (*p* < 0.0083, Figure 2A), we did not observe any further significant differences regarding the medium and long-term all-cause mortality (≥90 days) between these 2 groups (Figures 2B–E). In addition, patients in β-blockers + H2RAs group had significantly lower all-cause mortality from 30 days to 10 years as compared with patients in β-blockers group (*p* < 0.0083).

**FIGURE 3:**
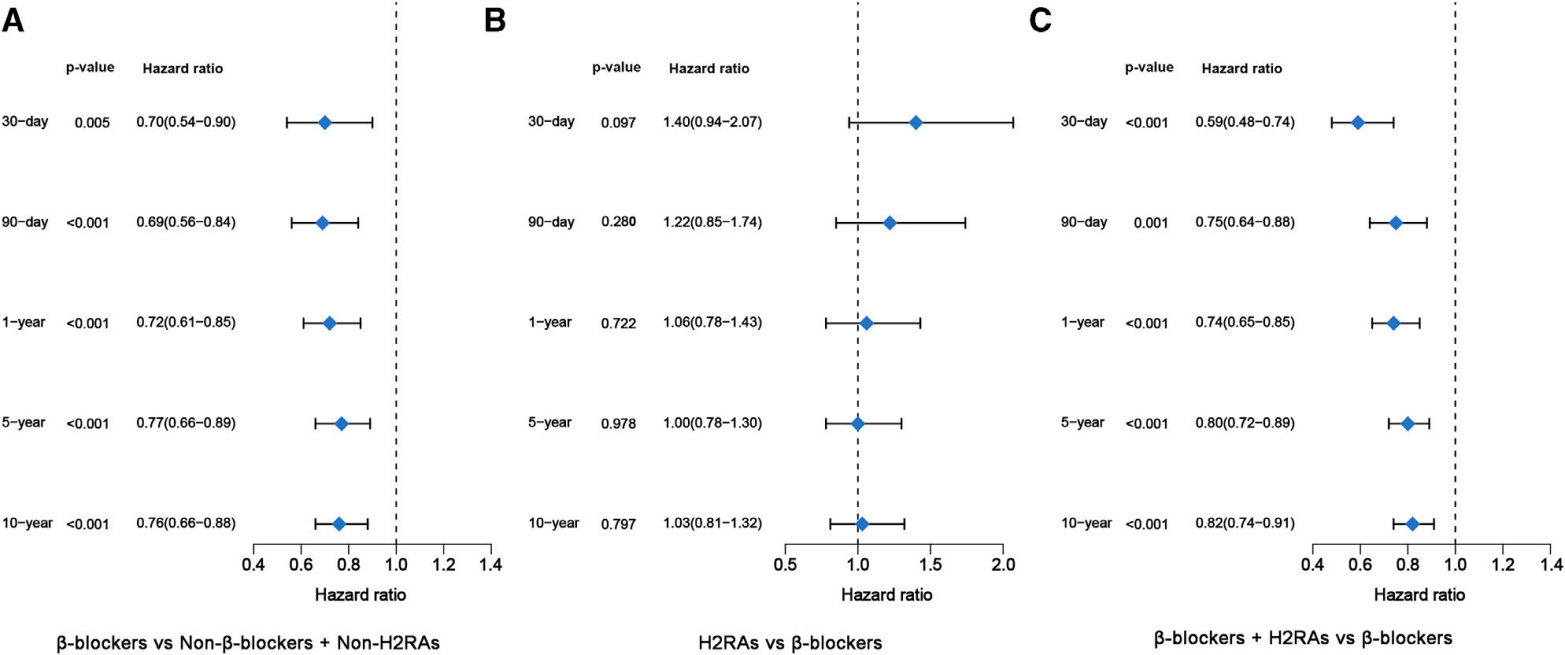
Forest plots of multivariate Cox regression model before PSM. **(A)** β-blockers group *versus* Non-β-blockers + Non-H2RAs group; **(B)** H2RAs group *versus* β-blockers group; **(C)** β-blockers + H2RAs *versus* β-blockers group.

Next, we performed further multivariate analyses using Cox regression models to evaluate the difference of long-term and short-term all-cause mortality among the 4 groups. In accordance with the above univariate analysis result, β-blockers exposure was also significantly associated with decreased all-cause mortality from 30 days to 10 years, respectively (*p* < 0.05, Figure 3A). Likewise, patients exposed to H2RAs had similar all-cause mortality with patients exposed to β-blockers (Figure 3B). Moreover, the use of β-blockers in conjunction with H2RAs was significantly superior to the use of β-blockers alone in reducing all-cause mortality of ICU patients with HF (Figure 3C).

Then, we analyzed the difference of secondary outcomes including ICU mortality, hospital mortality, ICU LOS and hospital LOS among the 4 groups, which showed that patients in β-blockers + H2RAs group had the lowest hospital and ICU mortality but the longest ICU and hospital LOS (Table 2). Moreover, patients of β-blockers group had lower ICU and hospital mortalities but longer ICU LOS than patients of Non-β-blockers + Non-H2RAs group (*p* < 0.0083). We also found that patients of H2RAs group had relatively higher ICU and hospital mortalities than of β-blockers group (*p* < 0.0083). However, the ICU and hospital LOS of patients exposed to H2RAs did not exhibit any significant difference with those of patients exposed to β-blockers.

**TABLE 2:** Secondary outcomes in four groups before matching.

	β-blockers +H2RAs	β-blockers	H2RAs	Non-β-blockers +Non-H2RAs
**Mortality, n (%)**				
ICU mortality	68 (3.3)^#^*	166 (6.6)*	24 (16.3)^#^	110 (13.0)
Hospital mortality	123 (5.9)^#^*	255 (10.1)*	31 (21.1)^#^	152 (18.0)
**Length of stay (day)**				
ICU LOS	3.0 (1.6–5.1)^#^*	2.3 (1.3–4.0)*	2.5 (1.6–4.9)	2.1 (1.2–4.1)
Hospital LOS	9.3 (6.2–14.3)^#^*	6.9 (4.3–11.2)	6.6 (4.6–9.5)	7.0 (3.9–11.2)

# < 0.0083 (0.05/6) *versus* β-blockers group after Bonferroni correction.

* *p* < 0.0083 (0.05/6) *versus* Non-β-blockers + Non-H2RAs, group after Bonferroni correction.

## Comparison of clinical outcomes of critical ill patients with HF between β-blockers and Non-β-blockers + Non-H2RAs group after propensity score matching

**FIGURE 4:**
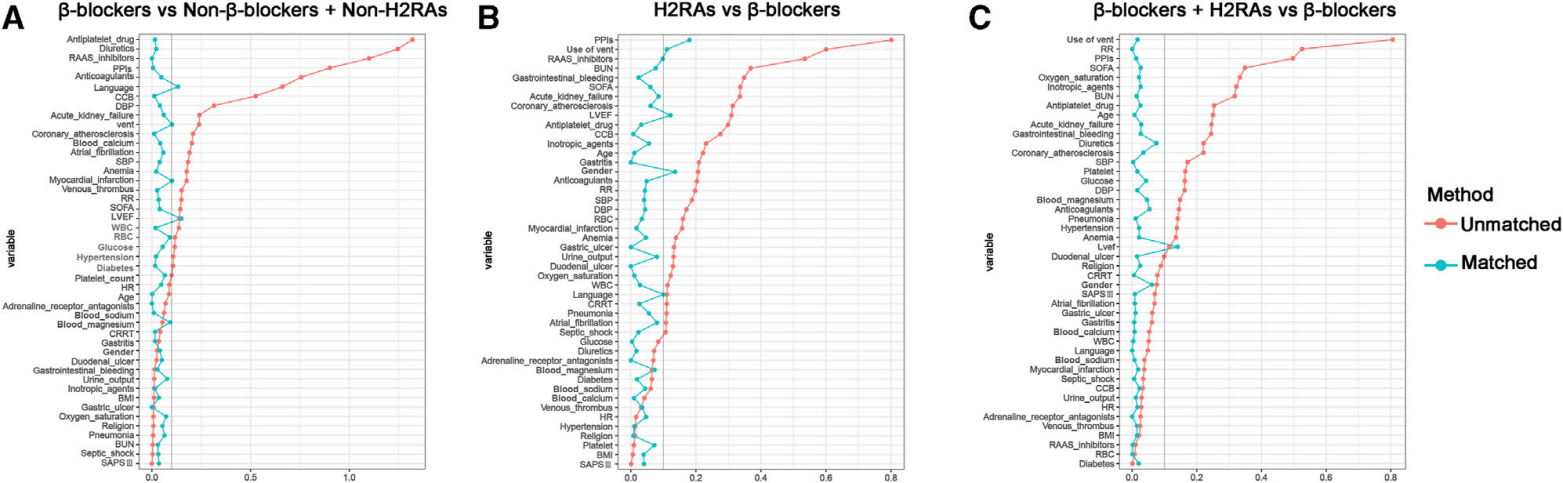
Standardized mean difference (SMD) of variables before and after propensity score matching. **(A)** β-blockers group *versus* Non-β-blockers + Non-H2RAs group; **(B)** H2RAs group *versus* β-blockers group; **(C)** β-blockers + H2RAs *versus* β-blockers group.

To further evaluate the effect of β-blockers on clinical outcomes of critically ill patients with HF, PSM was performed between Non-β-blockers + Non-H2RAs group and β-blockers group with ratio of 1:1. After PSM, 391 patients were included in each group and the baseline characteristics of the 2 groups were listed in Supplementary Table S3. The matching results showed that a majority of variables were with SMDs < 0.1, suggesting no major imbalances in the demographics and clinical characteristics after PSM (Figure 4A). Although certain variables (i.e., LVEF and language) exhibited relatively greater SMD values over 0.1, they did not exceed 0.2, which were also acceptable as moderate balanced according to previous reports (Zhang et al., 2019; Reijnders et al., 2022).

**FIGURE 5:**
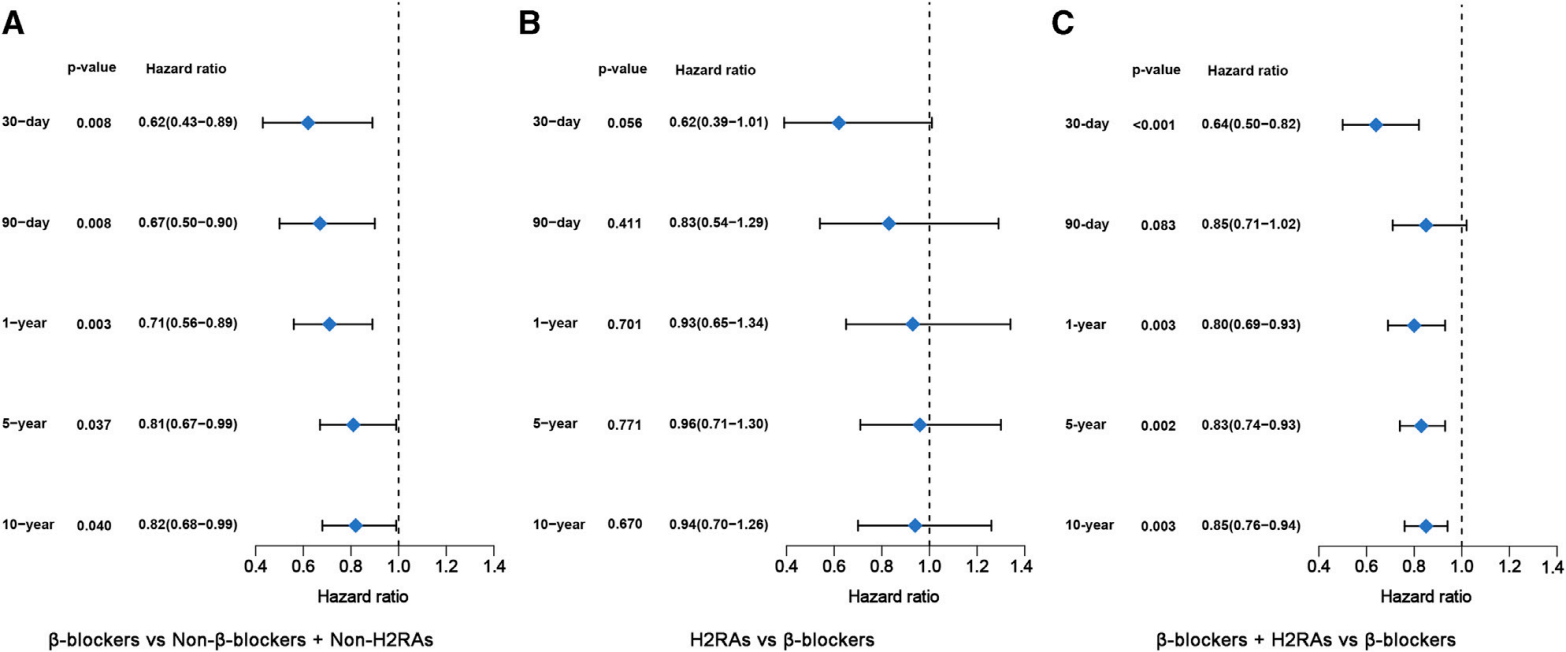
Forest plots of multivariate Cox regression model after PSM. **(A)** β-blockers group *versus* Non-β-blockers + Non-H2RAs group; **(B)** H2RAs group *versus* β-blockers group; **(C)** β-blockers + H2RAs *versus* β-blockers group.

For primary outcomes, the initial univariate analysis results showed that β-blockers exposure were significantly associated with reduced short- and medium-term (≤1-year) all-cause mortalities in ICU patients with HF (*p* < 0.01, Supplementary Figure S1). Conformably, the following multivariate also demonstrated that all kinds of all-cause mortalities were significantly reduced in β-blockers group after adjusting for the included covariates (*p* < 0.05, Figure 5A). As for secondary outcomes, the hospital mortality of β-blockers group was still lower than that of Non-β-blockers + Non-H2RAs group (Table 3). However, the hospital LOS of β-blockers group was longer than that of Non-β-blockers + Non-H2RAs group (Table 3).

**TABLE 3:** Secondary outcomes among four groups after matching.

	Mortality, n (%)				Length of stay (day)			
	ICU mortality	*p*-value	Hospital mortality	*p*-value	ICU LOS	*p*-value	Hospital LOS	*p*-value
**β-blockers vs. Non-β-blockers + Non-H2RAs**								
β-blockers group	39 (10.0)	0.120	57 (14.6)	0.002	2.2 (1.3–3.9)	0.204	6.8 (3.9–10.9)	0.014
Non-β-blockers + Non-H2RAs group	53 (13.6)		73 (18.7)		2.1 (1.2–3.6)		5.8 (3.3–10.0)	
**H2RAs vs. β-blockers**								
H2RAs group	18 (14.6)	0.025	24 (19.5)	0.008	4.1 (3.5–4.5)	0.684	6.8 (4.5–9.6)	0.874
β-blockers group	30 (7.8)		40 (10.4)		4.0 (3.5–4.4)		6.6 (4.0–11.3)	
**β-blockers + H2RAs vs. β-blockers**								
β-blockers + H2RAs group	40 (3.3)	<0.001	86 (7.1)	0.002	3.0 (1.7–5.5)	<0.001	9.8 (6.1–15.6)	<0.001
β-blockers group	94 (7.7)		129 (10.6)		2.4 (1.3–4.4)		7.1 (4.2–11.8)	

## Comparison of clinical outcomes of critically ill patients with HF between β-blockers and H2RAs group after propensity score matching

In this comparison, PSM was performed with ratio of 1:4. Eventually, H2RAs group included 123 patients and β-blockers group included 383 patients (Supplementary Table S4). The SMDs of most variables were < 0.1, with 4 exceptions (i.e., PPIs, use of vent, LVEF, and gender), indicating that the characteristics were moderately balanced between the comparison groups (Figure 4B). The univariate survival analyses showed that, although the short-term (≤30 days) all-cause mortalities of H2RAs users were significantly higher than those exposed with β-blockers (*p* = 0.0069), medium- and long-term (≥90 days) all-cause mortalities of patients exposed to H2RAs showed no significantly difference with those of patients exposed to β-blockers (Supplementary Figure S2). Furthermore, the multivariate Cox regression analysis results also indicated that the 2 groups had similar effect in reducing mortalities from 30 days to 10 years (Figure 5B). The comparison of secondary outcomes between the 2 groups showed that patents in β-blockers group had lower ICU and hospital mortalities than patents in H2RAs group (*p* < 0.05, Table 3).

## Comparison of clinical outcomes of critically ill patients with HF between β-blockers + H2RAs and β-blockers group after propensity score matching

After 1:1 PSM, 1219 pairs of patients were matched between the 2 groups and their baseline characteristics were summarized in Supplementary Table S5. Most covariates showed SMDs < 0.1 except LVEF (SMD = 0.121), confirming that the 2 groups were highly balanced for reliable downstream comparisons (Figure 4C).

Kaplan–Meier survival curves of all-cause mortality from 30 days to 10 years between these 2 groups after PSM were shown in Supplementary Figure S3. We found that use of β-blockers combined with H2RAs significantly reduced 30-day, 1-year, 5-year and 10-year all-cause mortality of critically ill patients with HF compared with β-blockers alone (*p* < 0.05). Furthermore, multivariate Cox regression analysis illustrated that 30-day, 1-year, 5-year and 10-year all-cause mortality of patients exposed to both β-blockers and H2RAs were significantly lower than those of patients only exposed to β-blockers (Figure 5C), which were in accordance with the Kaplan–Meier curves. Additionally, comparison of the secondary outcomes between the 2 groups after PSM showed that ICU and hospital mortalities of patients in β-blockers + H2RAs group were also significantly lower than those of patients in β-blockers group. However, ICU and hospital LOS of patients in β-blockers + H2RAs group were significantly longer than those of patients in β-blockers group (Table 3).

### Subgroup analysis

We next performed additional subgroup analyses stratified by gender among 4 groups and the results were shown in Supplementary Table S6. We observed that β-blockers exposure was significantly associated with decreased all-cause mortality from 30 days to 10 years among male HF patients but was only significantly associated with decreased 30- and 90-day mortality among female HF patients between Non-β-blockers + Non-H2RAs group and β-blockers group. For β-blockers *versus* H2RAs, it was found that the 2 groups exhibited similar effect in reducing mortalities from 30 days to 10 years in both sexes. As for β-blockers + H2RAs *versus* β-blockers, the use of β-blockers combined with H2RAs significantly reduced each kind of all-cause mortality compared with β-blockers alone among male HF patients, while these associations were not observed in short- and medium-term (≤1 year) among female HF patients. These findings indicated that male HF patients might be more sensitive to the use of H2RAs and β-blockers (either alone or in combination), which was similar to the previous report (Larson et al., 2022) and provided clues to warrant further population sensitivity studies regarding these 2 classes of drugs.

## Discussion

So far as we know, this large population-based cohort study is the first to compare the clinic outcomes of critically ill patients with HF exposed to either β-blockers or H2RAs. One strength of this study was that the impact of these 2 kinds of drugs on long-term mortality was studied. The relatively large sample size and more accurate grouping enabled the present comparison results more convincing and reliable. It was found that no significant difference in clinical outcomes was observed between these 2 kinds of drugs alone while that their combination had significantly lower mortality compared with β-blockers alone, which further suggested that β-blockers and H2RAs might have comparable efficacy in treating HF and demonstrated an additive or even synergistic effect between them. These findings provide more theoretical evidence for rational application of H2RAs and assessment of its therapeutic value in HF population.

One important observation of this study was that the use of β-blockers was a protective factor for HF patients and significantly reduced the all-cause mortality among them. Considering the status of β-blockers in HF treatment, this result was highly consistent with previous relevant consensus (Tsuyuki et al., 2000) as well as guideline recommendation (Heidenreich et al., 2022) and thus provided relatively fine evidence suggesting that the present research protocol was feasible and the population included in our study was representative. Furthermore, despite the relatively small population size in H2RAs group, it was still observed that the exposure of this kind of drugs had a tendency to reduce short- and medium-term all-cause mortality and was even significantly associated with decreased long-term all-cause mortality (Figure 2), which was also in accordance with our recent investigation (Huang et al., 2022) and further demonstrated the protective role of H2RAs in critically ill patients with HF. These control data formed a solid foundation for the following analyses of the present research.

In the comparative analyses, we provided novel evidence that H2RAs exposure was associated with similar decreased all-cause mortality as β-blockers exposure did, which demonstrated that H2RAs had parallel anti-HF effect with β-blockers, especially for medium- and long-term outcomes, and strongly indicated that H2RAs might be, at least in part, an ideal kind of substitution to β-blockers in the treatment of HF because of their common pharmacological effects (i.e., down-regulation of cAMP and negative chronotropic and inotropic effects). It is noteworthy that, although β-blockers are one of the key recommended treatments for HF currently, the intrinsic disadvantages of this kind of drugs inevitably lead to relatively low drug adherence and even a series of adverse events in clinical practice (Garcia et al., 2021; Azzouz et al., 2022). In this regard, H2RAs may have certain advantages over β-blockers. An important point is that most H2RAs are over-the-counter drugs in most countries with relatively low market price and moderately safe profile according to our previous evidence-based analysis (Meng et al., 2023), which provided a much safer potential treatment strategy for HF as compared with β-blockers especially considering that most chronic HF patients need long-term or even lifelong medication. Furthermore, although it is well acknowledged that β-blockers have good curative effect for patients with HFrEF, little evidence supports the benefits of β-blockers in HF with preserved ejection fraction (HFpEF) (Cleland et al., 2018; Meyer and Lewinter, 2019; Brinker et al., 2021). However, our previous study demonstrated that H2RAs improved survival of patients with both HFpEF and HFrEF (Huang et al., 2022), indicating that H2RAs might have certain potential to be applied to treat various types of HF. Additionally, oxidative stress, as one of the major causative factors of gastric ulcers, was well acknowledged to be significantly increased during HF (Sawyer, 2011; Zaghlool et al., 2019) and patients with HF are herein more prone to develop stress ulcers, which, luckily, is a main approved indication of H2RAs (but not β-blockers) in clinical practice. These advantages of H2RAs do provide a potential alternative candidate anti-HF strategy especially for those encountering intolerance of β-blockers during their HF treatment.

Nevertheless, it should be noted that, though not significant, exposure to β-blockers still exhibited a tendency to be associated with more decreased short- and medium-term (<1 year) all-cause mortality compared with H2RAs and that β-blockers were related to significantly lower 30-day all-cause mortality (Supplementary Figure S2) and ICU/hospital mortalities (Table 3) according to the present results, which suggested that β-blockers had relatively superior anti-HF effect than H2RAs for patients within short period after onset of sever HF. This might be an important advantage of β-blockers and patients with onset of HF less than 1 year, especially during hospital stay, are therefore still preferentially recommended to use β-blockers rather than H2RAs. Furthermore, we also observed that, with the follow-up time increased, the mortality-decreasing effect of H2RAs was gradually strengthened and exhibited a trend to be better than β-blockers when the follow-up time was over 1 year. Therefore, H2RAs may achieve better long-term benefits than β-blockers regarding their anti-HF effect and are hence worthy of further attention and exploration. However, the accuracy of the long-term survival results still deserved cautious interpretation and further validation as long-term survival data (especially for 5–10 years) in the present database may be more susceptible to various unknown factors.

Another key finding of the present study was that exposure to both β-blockers and H2RAs exhibited the strongest mortality-decreasing effect as compared with β-blockers or H2RAs used alone at each observed follow-up time point. This is quite reasonable as cardiac H2Rs and β1 receptors have long been demonstrated to have mutual synergistic effects upon activation according to our early fundamental investigation (He et al., 2012). In fact, we previously proved that histamine was a novel sympathetic neurotransmitter coexisted with norepinephrine (He et al., 2008; He et al., 2009; Hu et al., 2011) and exerted significant postsynaptic effects upon sympathetic overactivity (He et al., 2008; He et al., 2012). Therefore, considering that postsynaptic receptor synergism is a common phenomenon regarding β receptors (Smith and Burnstock, 2004; He et al., 2012; Kume et al., 2018; Rousseau et al., 2022), novel treatment strategy based on blockade of both H2Rs and β1 receptors is very likely to obtain a much better anti-HF effect with simultaneously decreased dosages of both drugs and, as a result, fewer adverse events although more clinical evidence is still required. In this regard, it may be a potential recommendation in future clinical practice to use H2RAs as an alternative to β-blockers for patients who are intolerant to β-blockers or in combination with β-blockers to increase efficacy and reduce adverse reactions of β-blockers although further prospective studies are needed to confirm the present findings.

As for the secondary outcomes, the present study still observed that the combined use of H2RAs and β-blockers had longer hospital and ICU LOS than β-blockers alone in critically ill patients with HF, which was in accordance with our recent study (Huang et al., 2022) and indicated that this might be a universal disadvantage of H2RAs exposure among this kind of patients. However, considering the significant association of combined exposing to H2RAs and β-blockers with largely reduced mortality of HF patients according to the present results, this minor disadvantage of H2RAs exposure would still be acceptable.

Several limitations of the present study should be mentioned. First, since this was a single center retrospective cohort study, selection bias was inevitable. Further multicenter-based prospective cohort studies are needed to confirm the present results. Second, despite having adjusted for confounders by PSM, unmeasured residual confounding factors still could not be completely excluded. Third, due to limitations in the public database, some lifestyle factors influencing survival and prognosis of patient with HF, such as dietary habits, alcohol drinking, smoking and pre-hospital prescriptions, were unable to be extracted. Fourth, the HF definition of the MIMIC III database is based on the ICD-9 disease codes, which might lead to bias in patient selection. Finally, specific types of H2RAs and β-blockers or different types of HF (HFpEF and HFrEF) may show different interactions, but the small sample size of the present H2RAs group limited further subgroup analysis. These limitations should be overcome in future well-designed studies.

In conclusion, the present study showed that H2RAs exposure exhibited comparable all-cause mortality-decreasing effect as β-blockers in critically ill patients with HF and that H2RAs and β-blockers had additive or synergistic interactions to improve survival of HF patients. These findings further supported the significant treatment value of H2RAs in patients with HF and offered an ideal potential alternative to β-blockers in future clinical practice.

## Data Availability

Publicly available datasets were analyzed in this study. This data can be found here: https://mimic.mit.edu/. The data that support the findings of this study are available on request from the corresponding author.
