# Supplementary material for: Histamine H2 receptor antagonist exhibited comparable all-cause mortality-decreasing effect as β-blockers in critically ill patients with heart failure: a cohort study
Source: Front Pharmacol. 2023 Nov 13;14:1273640. doi: 10.3389/fphar.2023.1273640 (PMC10683642; doi:10.3389/fphar.2023.1273640)
Supplement: Supplementary file 1 [file DataSheet1.ZIP › Supplemental materials/Supplemental materials.docx]

**Supplementary Figure S1.** Kaplan-Meier survival curves of β-blockers group and Non-β-blockers + Non H2RAs group after matching. (A) 30-day mortality; (B) 90-day mortality; (C) 1-year mortality; (D) 5-year mortality; (E) 10-year mortality.

**Supplementary Figure S2.** Kaplan-Meier survival curves of H2RAs group and β-blockers group after matching. (A) 30-day mortality; (B) 90-day mortality; (C) 1-year mortality; (D) 5-year mortality; (E) 10-year mortality.

**Supplementary Figure S3.** Kaplan-Meier survival curves of β-blockers + H2RAs group and β-blockers group after matching. (A) 30-day mortality; (B) 90-day mortality; (C) 1-year mortality; (D) 5-year mortality; (E) 10-year mortality.
