# Supplementary material for: Histamine H2 receptor antagonist exhibited comparable all-cause mortality-decreasing effect as β-blockers in critically ill patients with heart failure: a cohort study
Source: Front Pharmacol. 2023 Nov 13;14:1273640. doi: 10.3389/fphar.2023.1273640 (PMC10683642; doi:10.3389/fphar.2023.1273640)
Supplement: Supplementary file 1 [file DataSheet1.ZIP › Supplemental materials/Supplementary Table S3.docx]

| **Supplementary Table S3 Baseline characteristics β-blockers and Non-β-blockers + Non-H2RAs after matching** | | | | |
| --- | --- | --- | --- | --- |
|  | **β-blockers**  **(n=391)** | **Non-β-blockers + Non-H2RAs (n=391)** | **P-value** | **SMD** |
| Age, years | 74.72 ± 13.90 | 73.62 ± 14.40 | 0.278 | 0.078 |
| Gender, female, n (%) | 206 (52.7) | 199 (50.9) | 0.668 | 0.036 |
| BMI, kg/m^2^ | 28.38 ± 6.93 | 29.03 ± 7.83 | 0.218 | 0.088 |
| SOFA | 4.26 ± 2.60 | 4.17 ± 2.97 | 0.654 | 0.032 |
| SAPSⅢ | 47.50 ± 18.04 | 46.06 ± 20.13 | 0.293 | 0.075 |
| CRRT, n (%) | 10 (2.6) | 11 (2.8) | 1 | 0.016 |
| Use of ventilator, n (%) | 122 (31.2) | 126 (32.2) | 0.818 | 0.022 |
| Language, English, n (%) | 219 (56.0) | 203 (51.9) | 0.282 | 0.139 |
| Religion, Catholic, n (%) | 143 (36.6) | 129 (33.0) | 0.294 | 0.005 |
| Vital signs |  |  |  |  |
| HR | 80.87 ± 20.97 | 80.20 ± 20.47 | 0.650 | 0.032 |
| SBP, mmHg | 121.80 ± 24.79 | 121.87 ± 23.94 | 0.964 | 0.003 |
| DBP, mmHg | 60.56 ± 15.80 | 60.60 ± 15.85 | 0.969 | 0.003 |
| Oxygen saturation, (%) | 96.38 ± 3.85 | 96.53 ± 3.80 | 0.594 | 0.038 |
| RR | 20.10 ± 5.67 | 19.69 ± 5.84 | 0.317 | 0.072 |
| Laboratory parameters |  |  |  |  |
| RBC, m/μL | 3.70 (3.30-4.28) | 3.76 (3.25-4.23) | 0.915 | 0.098 |
| WBC, k/μL | 10.0 (7.3-3.9) | 9.6 (7.2-13.5) | 0.477 | 0.051 |
| Platelet count, k/μL | 230 (177-297) | 224 (170-293) | 0.938 | 0.006 |
| Glucose, mg/dL | 128 (104-165) | 126 (105-160) | 0.189 | 0.094 |
| Blood sodium, mEq/L | 138 (135-141) | 139 (135-141) | 0.437 | 0.056 |
| Blood magnesium, mg/dL | 2.0 (1.8-2.2) | 2.0 (1.8-2.3) | 0.464 | 0.092 |
| Blood calcium, mg/dL | 8.7 (8.1-9.1) | 8.7 (8.2-9.1) | 0.903 | 0.009 |
| BUN, mg/dL | 28 (19-51) | 27 (17-47) | 0.587 | 0.039 |
| urine output, mL | 1.6 (1.0-2.4) | 1.8 (1.0-2.7) | 0.107 | 0.093 |
| LVEF, n (%) |  |  | 0.277 | 0.141 |
| 10–35% | 105 (26.9) | 87 (22.3) |  |  |
| 35–55% | 220 (56.3) | 232 (59.3) |  |  |
| 55–70% | 43 (11.0) | 54 (13.8) |  |  |
| >70% | 23 (5.9) | 18 (4.6) |  |  |
| Co-morbidities, n (%) |  |  |  |  |
| Atrial fibrillation | 161 (41.2) | 145 (37.1) | 0.272 | 0.084 |
| Myocardial infarction | 53 (13.6) | 39 (10.0) | 0.149 | 0.100 |
| Coronary atherosclerosis | 139 (35.5) | 114 (29.2) | 0.067 | 0.003 |
| Hypertension | 150 (38.4) | 145 (37.1) | 0.768 | 0.026 |
| Venous thrombosis | 15 (3.8) | 14 (3.6) | 1 | 0.014 |
| Anemia | 134 (34.3) | 130 (33.2) | 0.821 | 0.022 |
| Pneumonia | 84 (21.5) | 83 (21.2) | 1 | 0.006 |
| Diabetes | 137 (35.0) | 126 (32.2) | 0.449 | 0.060 |
| Duodenal ulcer | 4 (1.0) | 3 (0.8) | 1 | 0.027 |

**Supplementary Table S3 Continued**

|  | **β-blockers**  **(n=391)** | **Non-β-blockers + Non-H2RAs**  **(n=391)** | **P-value** | **SMD** |
| --- | --- | --- | --- | --- |
| Gastric ulcer | 5 (1.3) | 3 (0.8) | 0.722 | 0.051 |
| Gastrointestinal bleeding | 30 (7.7) | 28 (7.2) | 0.891 | 0.020 |
| Gastritis | 11 (2.8) | 10 (2.6) | 1 | 0.016 |
| Acute kidney failure | 144 (36.8) | 131 (33.5) | 0.369 | 0.070 |
| Septic shock | 11 (2.8) | 9 (2.3) | 0.821 | 0.032 |
| Medications, n (%) |  |  |  |  |
| RAAS inhibitors | 139 (35.5) | 131 (33.5) | 0.599 | 0.043 |
| Diuretics | 266 (68.0) | 280 (71.6) | 0.311 | 0.078 |
| Inotropic agents | 160 (40.9) | 139 (35.5) | 0.141 | 0.011 |
| Adrenaline receptor antagonist | 391 (100.0) | 391 (100.0) | - | <0.001 |
| CCB | 96 (24.6) | 93 (23.8) | 0.867 | 0.018 |
| PPIs | 254 (65.0) | 262 (67.0) | 0.597 | 0.043 |
| Anticoagulants | 327 (83.6) | 327 (83.6) | 1 | <0.001 |
| Antiplatelet drugs | 186 (47.6) | 190 (48.6) | 0.830 | 0.020 |

Abbreviations: H2RA, histamine H2 receptor antagonist; SMD, standardized mean difference; BMI, body mass index; SOFA, sequential organ failure assessment score; SAPSⅢ, simplified acute physiology score Ⅲ; CRRT, Continuous renal replacement therapy; HR, heart rate; SBP, systolic blood pressure; DBP, diastolic blood pressure; RR, respiratory rate; WBC, white blood cell; RBC, red blood cell; BUN, blood urea nitrogen; LVEF, left ventricular ejection fraction; RAAS, renin angiotensin aldosterone system; CCB, calcium channel blockers; PPIs, proton pump inhibitors; ICU, indicates intensive care unit; LOS, length of stay.
