# Supplementary material for: Histamine H2 receptor antagonist exhibited comparable all-cause mortality-decreasing effect as β-blockers in critically ill patients with heart failure: a cohort study
Source: Front Pharmacol. 2023 Nov 13;14:1273640. doi: 10.3389/fphar.2023.1273640 (PMC10683642; doi:10.3389/fphar.2023.1273640)
Supplement: Supplementary file 1 [file DataSheet1.ZIP › Supplemental materials/Supplementary Table S4.docx]

| **Supplementary Table S4 Baseline characteristics of H2RAs group and β-blockers group after matching** | | | | |
| --- | --- | --- | --- | --- |
|  | **H2RAs**  **(n=123)** | **β-blockers**  **(n=383)** | **P-value** | **SMD** |
| Age, years | 71.94 ± 15.45 | 72.98 ± 13.72 | 0.479 | 0.071 |
| Gender, female, n (%) | 70 (56.9) | 210 (54.8) | 0.765 | 0.124 |
| BMI, kg/m^2^ | 28.76 ± 6.79 | 28.54 ± 7.15 | 0.761 | 0.032 |
| SOFA | 4.54 ± 3.03 | 4.55 ± 2.95 | 0.984 | 0.002 |
| SAPSⅢ | 45.28 ± 19.85 | 45.51 ± 17.70 | 0.907 | 0.012 |
| CRRT, n (%) | 3 (2.4) | 10 (2.6) | 1 | 0.011 |
| Use of ventilator, n (%) | 61 (49.6) | 172 (44.9) | 0.422 | 0.114 |
| Language, English, n (%) | 46 (37.4) | 142 (37.1) | 1 | 0.007 |
| Religion, Catholic, n (%) | 46 (37.3) | 153 (39.9) | 0.296 | 0.001 |
| Vital signs |  |  |  |  |
| HR | 82.50 ± 20.69 | 80.57 ± 21.22 | 0.378 | 0.092 |
| SBP, mmHg | 120.92 ± 22.20 | 122.69 ± 25.78 | 0.494 | 0.074 |
| DBP, mmHg | 61.96 ± 15.16 | 62.10 ± 16.17 | 0.935 | 0.009 |
| Oxygen saturation, (%) | 97.21 ± 3.37 | 96.93 ± 3.39 | 0.418 | 0.084 |
| RR | 19.14 ± 6.20 | 19.20 ± 5.44 | 0.914 | 0.011 |
| Laboratory parameters |  |  |  |  |
| RBC, m/μL | 4.1 (3.5-4.5) | 4.0 (3.5-4.4) | 0.630 | 0.049 |
| WBC, k/μL | 10.0 (7.3-12.9) | 9.6 (7.2-14.0) | 0.829 | 0.022 |
| platelet count, k/μL | 249 (170-309) | 236 (183-298) | 0.964 | 0.005 |
| Glucose, mg/dL | 129 (97-175) | 130 (107-181) | 0.718 | 0.037 |
| blood sodium, mEq/L | 139 (136-141) | 139 (136-141) | 0.959 | 0.005 |
| blood magnesium, mg/dL | 2.0 (1.8-2.3) | 2.0 (1.8-2.2) | 0.926 | 0.010 |
| blood calcium, mg/dL | 8.9 (8.4-9.2) | 8.8 (8.3-9.2) | 0.861 | 0.019 |
| BUN, mg/dL | 22 (16-37) | 25 (17-34) | 0.644 | 0.047 |
| urine output, L | 1.6 (1.0-2.2) | 1.7 (1.0-2.5) | 0.444 | 0.081 |
| LVEF, n (%) |  |  | 0.740 | 0.117 |
| 10–35% | 31 (25.2) | 115 (30.0) |  |  |
| 35–55% | 66 (53.7) | 192 (50.1) |  |  |
| 55–70% | 18 (14.6) | 49 (12.8) |  |  |
| >70% | 8 (6.5) | 27 (7.0) |  |  |
| Co-morbidities, n (%) |  |  |  |  |
| Atrial fibrillation | 46 (37.4) | 149 (38.9) | 0.848 | 0.031 |
| Myocardial infarction | 15 (12.2) | 51 (13.3) | 0.867 | 0.034 |
| Coronary atherosclerosis | 39 (31.7) | 130 (33.9) | 0.728 | 0.048 |
| Hypertension | 54 (43.9) | 160 (41.8) | 0.756 | 0.043 |
| Venous thrombosis, | 6 (4.9) | 16 (4.2) | 0.938 | 0.034 |
| Anemia | 37 (30.1) | 106 (27.7) | 0.689 | 0.053 |
| Pneumonia | 27 (22.0) | 82 (21.4) | 0.999 | 0.013 |
| Diabetes | 44 (35.8) | 124 (32.4) | 0.558 | 0.072 |
| Duodenal ulcer | 123 (100.0) | 383 (100.0) | - | <0.001 |
| **Supplementary Table S4 Continued** | | | | |
|  | **H2RAs**  **(n=123)** | **β-blockers**  **(n=383)** | **P-value** | **SMD** |
| Gastric ulcer | 123 (100.0) | 383 (100.0) | - | <0.001 |
| Gastrointestinal bleeding | 1 (0.8) | 6 (1.6) | 0.858 | 0.070 |
| Gastritis | 123 (100.0) | 383 (100.0) | - | <0.001 |
| Gastric ulcer | 123 (100.0) | 383 (100.0) | - | <0.001 |
| Acute kidney failure | 28 (22.8) | 107 (27.9) | 0.312 | 0.080 |
| Septic shock | 4 (3.3) | 12 (3.1) | 1 | 0.007 |
| Medications, n (%) |  |  |  |  |
| RAAS inhibitors | 51 (41.5) | 190 (49.6) | 0.142 | 0.104 |
| Diuretics | 108 (87.8) | 341 (89.0) | 0.833 | 0.038 |
| Inotropic agents | 57 (46.3) | 185 (48.3) | 0.783 | 0.039 |
| Adrenaline receptor antagonist | 123 (100.0) | 383 (100.0) | - | <0.001 |
| CCB | 28 (22.8) | 98 (25.6) | 0.610 | 0.066 |
| PPIs | 55 (44.7) | 202 (52.7) | 0.148 | 0.161 |
| Anticoagulants | 105 (85.4) | 336 (87.7) | 0.599 | 0.069 |
| Antiplatelet drugs | 78 (63.4) | 267 (69.7) | 0.233 | 0.134 |

Abbreviations: H2RA, histamine H2 receptor antagonist; SMD, standardized mean difference; BMI, body mass index; SOFA, sequential organ failure assessment score; SAPSⅢ, simplified acute physiology score Ⅲ; CRRT, Continuous renal replacement therapy; HR, heart rate; SBP, systolic blood pressure; DBP, diastolic blood pressure; RR, respiratory rate; WBC, white blood cell; RBC, red blood cell; BUN, blood urea nitrogen; LVEF, left ventricular ejection fraction; RAAS, renin angiotensin aldosterone system; CCB, calcium channel blockers; PPIs, proton pump inhibitors; ICU, indicates intensive care unit; LOS, length of stay.
