# Supplementary material for: Histamine H2 receptor antagonist exhibited comparable all-cause mortality-decreasing effect as β-blockers in critically ill patients with heart failure: a cohort study
Source: Front Pharmacol. 2023 Nov 13;14:1273640. doi: 10.3389/fphar.2023.1273640 (PMC10683642; doi:10.3389/fphar.2023.1273640)
Supplement: Supplementary file 1 [file DataSheet1.ZIP › Supplemental materials/Supplementary Table S5.docx]

| **Supplementary Table S5 Baseline characteristics of β-blockers + H2RAs and β-blockers group after matching** | | | | |
| --- | --- | --- | --- | --- |
|  | **β-blockers + H2RAs**  **(n=1219)** | **β-blockers**  **(n=1219)** | **P-value** | **SMD** |
| Age, years | 72.66 ± 13.00 | 72.92 ± 13.82 | 0.630 | 0.020 |
| Gender, female, n (%) | 562 (46.1) | 543 (44.5) | 0.464 | 0.031 |
| BMI, kg/m^2^ | 28.78 ± 6.88 | 28.74 ± 6.72 | 0.870 | 0.007 |
| SOFA | 4.15 ± 2.65 | 4.18 ± 2.68 | 0.796 | 0.010 |
| SAPSⅢ | 44.74 ± 17.60 | 44.87 ± 17.51 | 0.852 | 0.008 |
| CRRT，n (%) | 23 (1.9) | 27 (2.2) | 0.668 | 0.023 |
| Use of ventilator, n (%) | 530 (43.5) | 519 (42.6) | 0.683 | 0.018 |
| Language, English, n (%) | 524 (43.0) | 543 (44.5) | 0.462 | 0.031 |
| Religion, Catholic, n (%) | 462 (37.8) | 494 (40.5) | 0.184 | 0.030 |
| Vital signs |  |  |  |  |
| HR | 81.53 ± 20.06 | 82.26 ± 20.97 | 0.375 | 0.036 |
| SBP, mmHg | 123.70 ± 24.40 | 123.50 ± 25.30 | 0.844 | 0.008 |
| DBP, mmHg | 63.38 ± 16.09 | 63.70 ± 16.75 | 0.639 | 0.019 |
| Oxygen saturation, (%) | 97.09 ± 3.41 | 97.16 ± 3.32 | 0.605 | 0.021 |
| RR | 18.89 ± 5.74 | 19.09 ± 5.55 | 0.401 | 0.034 |
| Laboratory parameters |  |  |  |  |
| RBC, m/μL | 3.95 (3.49-4.43) | 3.95 (3.49-4.43) | 0.637 | 0.019 |
| WBC, k/μL | 9.7 (7.2-13.0) | 9.5 (7.3-13.2) | 0.837 | 0.008 |
| Platelet count, k/μL | 228 (179-291) | 230 (181-295) | 0.796 | 0.010 |
| Glucose, mg/dL | 134 (109-176) | 130 (107-176) | 0.365 | 0.037 |
| Blood sodium, mEq/L | 138 (136-141) | 138 (136-141) | 0.865 | 0.007 |
| Blood magnesium, mg/dL | 2.0 (1.8-2.2) | 2.0 (1.8-2.2) | 0.379 | 0.036 |
| Blood calcium, mg/dL | 8.8 (8.3-9.2) | 8.8 (8.4-9.2) | 0.779 | 0.011 |
| BUN, mg/dL | 25 (18-39) | 26 (18-39) | 0.858 | 0.007 |
| urine output, L | 1.8 (1.1-2.6) | 1.8 (1.1-2.7) | 0.670 | 0.017 |
| LVEF, n (%) |  |  | 0.031 | 0.121 |
| 10–35% | 411 (33.7) | 445 (36.5) |  |  |
| 35–55% | 666 (54.6) | 598 (49.1) |  |  |
| 55–70% | 104 (8.5) | 133 (10.9) |  |  |
| >70% | 38 (3.1) | 43 (3.5) |  |  |
| Co-morbidities, n (%) |  |  |  |  |
| Atrial fibrillation | 559 (45.9) | 556 (45.6) | 0.935 | 0.005 |
| Myocardial infarction | 247 (20.3) | 238 (19.5) | 0.685 | 0.018 |
| Coronary atherosclerosis | 613 (50.3) | 635 (52.1) | 0.395 | 0.036 |
| Hypertension | 541 (44.4) | 533 (43.7) | 0.775 | 0.013 |
| Venous thrombosis, | 64 (5.3) | 60 (4.9) | 0.782 | 0.015 |
| Anemia | 376 (30.8) | 355 (29.1) | 0.377 | 0.038 |
| Pneumonia | 208 (17.1) | 217 (17.8) | 0.669 | 0.019 |
| Diabetes | 468 (38.4) | 468 (38.4) | 1 | <0.001 |
| Duodenal ulcer | 3 (0.2) | 3 (0.2) | 1 | <0.001 |
| **Supplementary Table S5 Continued** | | | | |
|  | **β-blockers + H2RAs**  **(n=1219)** | **β-blockers**  **(n=1219)** | **P-value** | **SMD** |
| Gastritis ulcer | 5 (0.4) | 8 (0.7) | 0.578 | 0.034 |
| Gastrointestinal bleeding | 42 (3.4) | 46 (3.8) | 0.745 | 0.018 |
| Gastritis | 21 (1.7) | 20 (1.6) | 1 | 0.006 |
| Acute kidney failure | 401 (32.9) | 392 (32.2) | 0.729 | 0.016 |
| Septic shock | 22 (1.8) | 24 (2.0) | 0.882 | 0.012 |
| Medications, n (%) |  |  |  |  |
| RAAS inhibitors | 789 (64.7) | 774 (63.5) | 0.554 | 0.026 |
| Diuretics | 1090 (89.4) | 1069 (87.7) | 0.203 | 0.054 |
| Inotropic agents | 572 (46.9) | 558 (45.8) | 0.598 | 0.023 |
| Adrenaline receptor antagonist | 3 (0.2) | 5 (0.4) | 0.723 | 0.029 |
| CCB | 403 (33.1) | 396 (32.5) | 0.796 | 0.012 |
| PPIs | 813 (66.7) | 819 (67.2) | 0.830 | 0.010 |
| Anticoagulants | 1109 (91.0) | 1096 (89.9) | 0.408 | 0.036 |
| Antiplatelet drugs | 994 (81.5) | 1008 (82.7) | 0.492 | 0.030 |

Abbreviations: H2RA, histamine H2 receptor antagonist; SMD, standardized mean difference; BMI, body mass index; SOFA, sequential organ failure assessment score; SAPSⅢ, simplified acute physiology score Ⅲ; CRRT, Continuous renal replacement therapy; HR, heart rate; SBP, systolic blood pressure; DBP, diastolic blood pressure; RR, respiratory rate; WBC, white blood cell; RBC, red blood cell; BUN, blood urea nitrogen; LVEF, left ventricular ejection fraction; RAAS, renin angiotensin aldosterone system; CCB, calcium channel blockers; PPIs, proton pump inhibitors; ICU, indicates intensive care unit; LOS, length of stay.
