# Supplementary material for: Histamine H2 receptor antagonist exhibited comparable all-cause mortality-decreasing effect as β-blockers in critically ill patients with heart failure: a cohort study
Source: Front Pharmacol. 2023 Nov 13;14:1273640. doi: 10.3389/fphar.2023.1273640 (PMC10683642; doi:10.3389/fphar.2023.1273640)
Supplement: Supplementary file 1 [file DataSheet1.ZIP › Supplemental materials/Supplementary Table S6.docx]

**Supplementary Table S6 Subgroup analyses stratified by gender among four groups after matching**

|  | **30-day** **all-mortality**  HR: (95%CI); P-value | **90-day all-mortality**  HR: (95%CI); P-value | **1-year all-mortality**  HR: (95%CI); P-value | **5-year all-mortality**  HR: (95%CI); P-value | **10-year all-mortality**  HR: (95%CI); P-value |
| --- | --- | --- | --- | --- | --- |
| **β-blockers vs**  **Non-β-blockers+Non-H2RAs** |  |  |  |  |  |
| Male (n=377) | 0.502 (0.295-0.854); 0.011 | 0.483 (0.314-0.745); 0.001 | 0.648 (0.464-0.905); 0.011 | 0.721 (0.537-0.967); 0.029 | 0.725 (0.546-0.964); 0.027 |
| Female (n=405) | 0.528 (0.302-0.923); 0.025 | 0.580 (0.369-0.912); 0.018 | 0.733 (0.524-1.025); 0.070 | 0.923 (0.699-1.219); 0.572 | 0.961 (0.737-1.252); 0.766 |
|  |  |  |  |  |  |
| **H2RAs vs β-blockers** |  |  |  |  |  |
| Male (n=226) | 1.688 (0.650-4.383); 0.283 | 1.357 (0.655-2.811); 0.411 | 1.022 (0.534-1.956); 0.947 | 0.854 (0.497-1.467); 0.567 | 0.913 (0.543-1.537); 0.732 |
| Female (n=280) | 1.813 (0.820-4.009); 0.142 | 1.398 (0.695-2.813); 0.347 | 1.217 (0.704-2.104); 0.482 | 1.047 (0.667-1.641); 0.843 | 1.028 (0.670-1.575); 0.961 |
|  |  |  |  |  |  |
| **β-blockers+H2RAs vs β-blockers** |  |  |  |  |  |
| Male (n=1333) | 0.582 (0.412-0.821); 0.002 | 0.716 (0.551-0.932); 0.013 | 0.682 (0.555-0.839); < 0.001 | 0.792 (0.673-0.933); 0.005 | 0.816 (0.698-0.955); 0.011 |
| Female (n=1105) | 0.784 (0.547-1.124); 0.185 | 1.010 (0.766-1.331); 0.946 | 0.903 (0.729-1.118); 0.348 | 0.839 (0.709-0.995); 0.043 | 0.847 (0.719-0.998); 0.047 |
