# Supplementary figures and images for: Histamine H2 receptor antagonist exhibited comparable all-cause mortality-decreasing effect as β-blockers in critically ill patients with heart failure: a cohort study
Source: Front Pharmacol. 2023 Nov 13;14:1273640. doi: 10.3389/fphar.2023.1273640 (PMC10683642; doi:10.3389/fphar.2023.1273640)

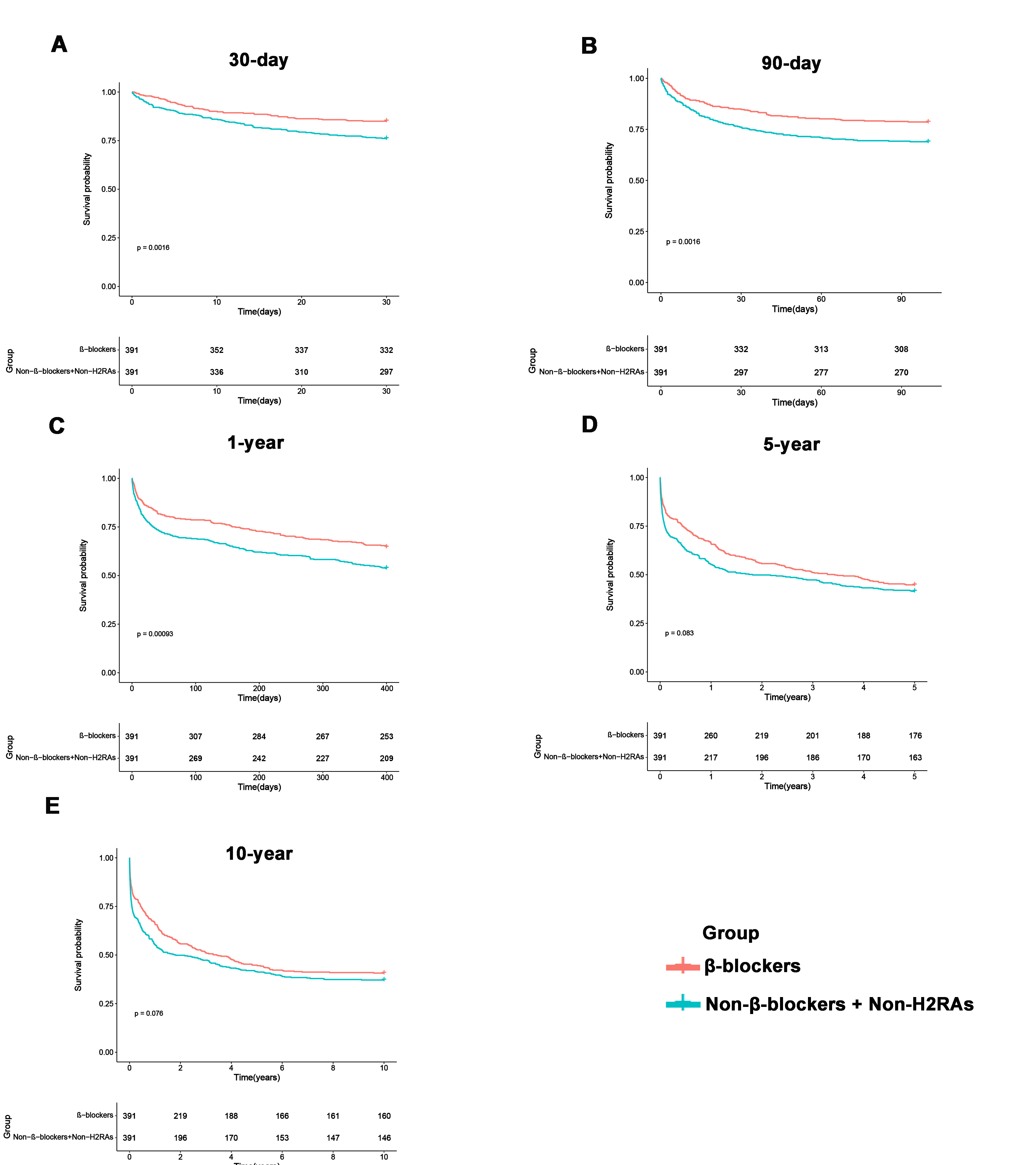

Supplement: Supplementary file 1 [file DataSheet1.ZIP › Supplemental materials/Supplementary Figure S1.tif]

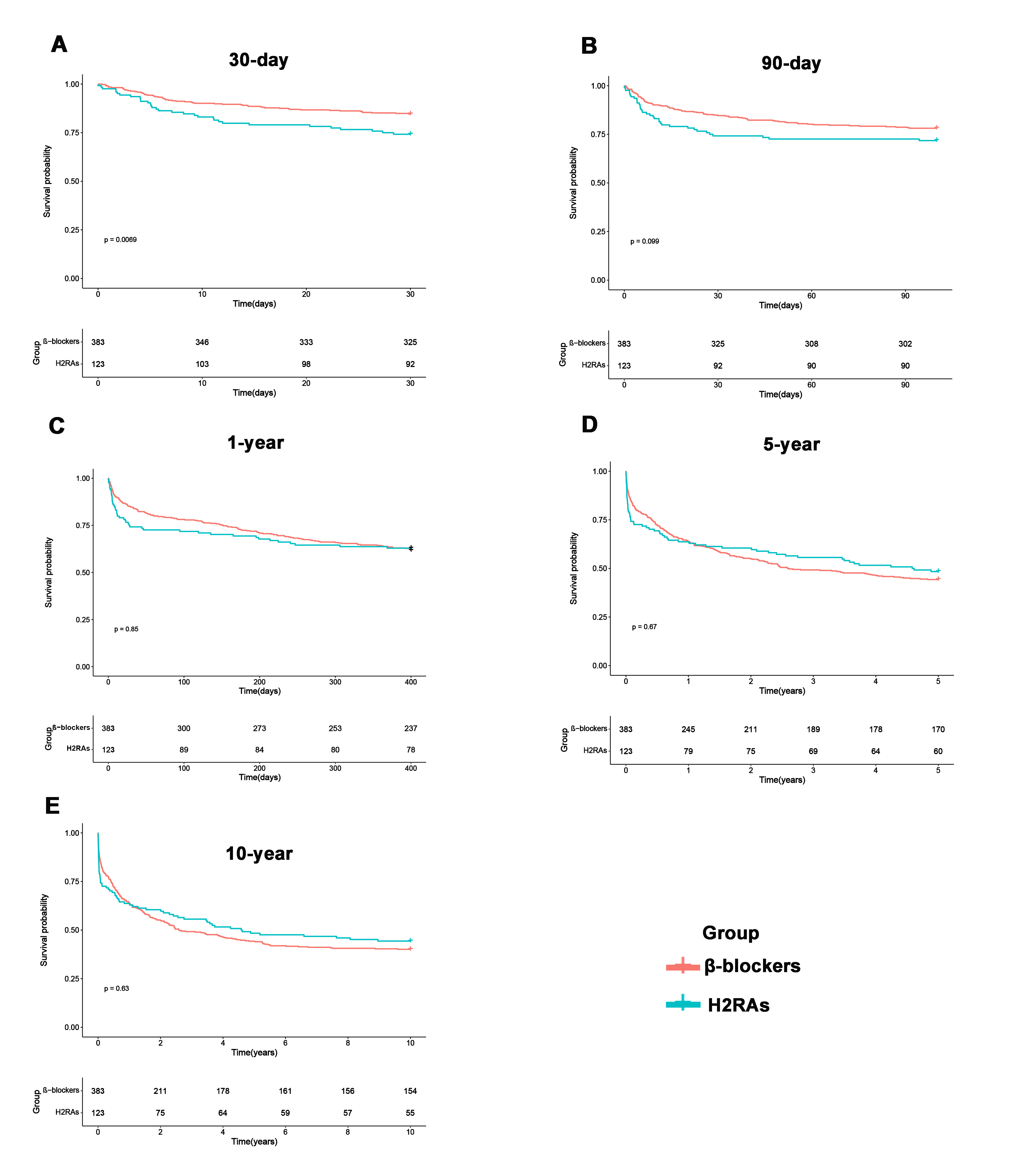

Supplement: Supplementary file 1 [file DataSheet1.ZIP › Supplemental materials/Supplementary Figure S2.tif]

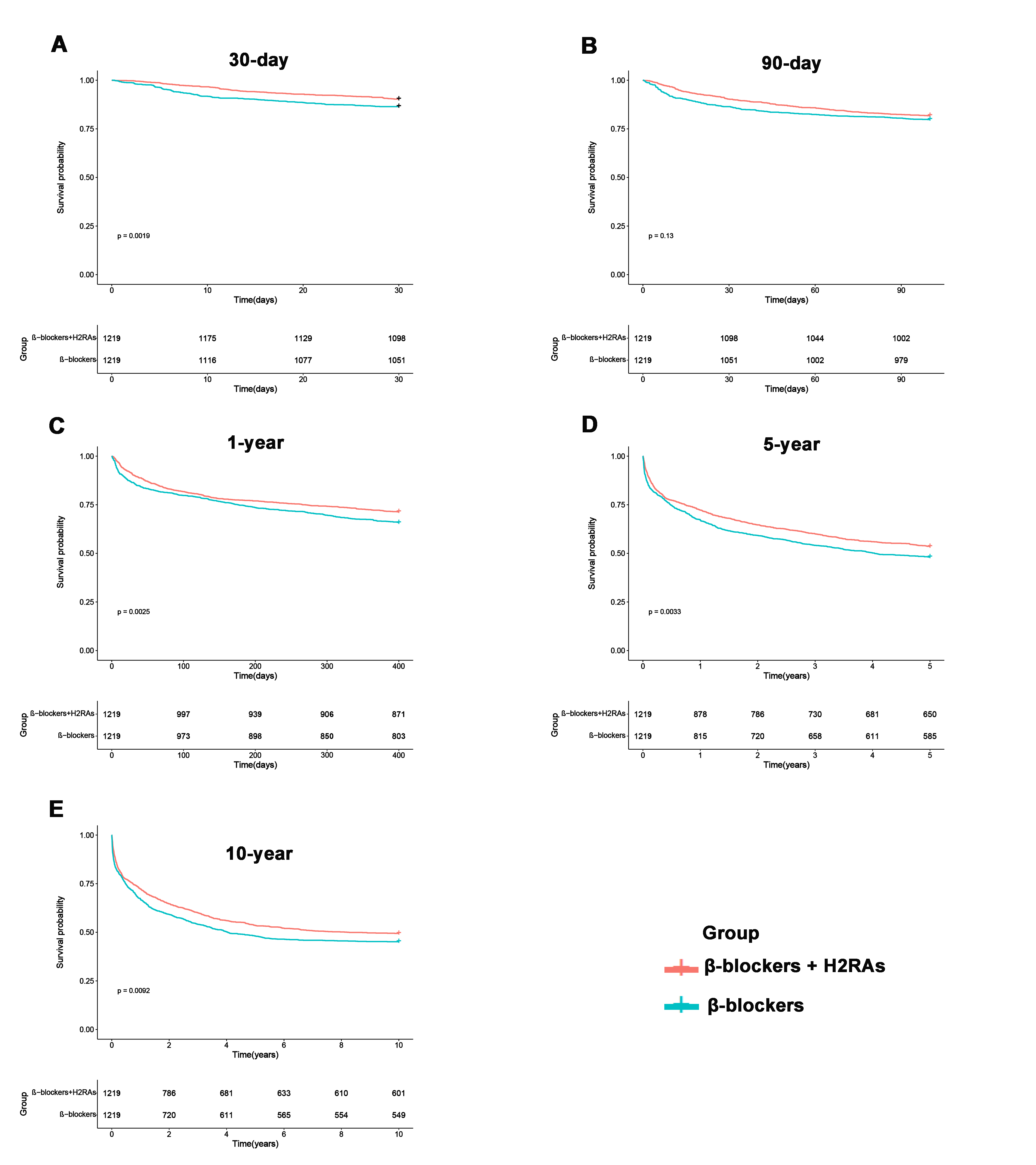

Supplement: Supplementary file 1 [file DataSheet1.ZIP › Supplemental materials/Supplementary Figure S3.tif]
