# Supplementary material for: Histamine H2 receptor antagonist exhibited comparable all-cause mortality-decreasing effect as β-blockers in critically ill patients with heart failure: a cohort study
Source: Front Pharmacol. 2023 Nov 13;14:1273640. doi: 10.3389/fphar.2023.1273640 (PMC10683642; doi:10.3389/fphar.2023.1273640)
Supplement: Supplementary file 1 [file DataSheet1.ZIP › Supplemental materials/Supplementary Table S2.doc]

Supplementary Table S2 Missing number (%) for included variables in the dataset

Abbreviations: SOFA, sequential organ failure assessment score; SAPSⅢ, simplified acute physiology score Ⅲ; CRRT, Continuous renal replacement therapy; HR, heart rate; SBP, systolic blood pressure; DBP, diastolic blood pressure; RR, respiratory rate; WBC, white blood cell; RBC, red blood cell; BUN, blood urea nitrogen; LVEF, left ventricular ejection fraction; RAAS, renin angiotensin aldosterone system; CCB, calcium channel blockers; PPIs, proton pump inhibitors; ICU, intensive care unit; LOS, length of stay.

| Variables | Missing, N (%) |
| --- | --- |
| Age | 0 (0) |
| Gender | 0 (0) |
| Height | 593 (10.6) |
| Weight | 101 (1.8) |
| SOFA | 0 (0) |
| SAPSIII | 0(0) |
| CRRT | 0(0) |
| Use of ventilator | 0 (0) |
| Language | 0 (0) |
| Religion | 0 (0) |
| HR | 82 (1.5) |
| SBP | 102 (1.8) |
| DBP | 72 (1.3) |
| Oxygen saturation | 112 (2.0) |
| RR | 90 (1.6) |
| RBC | 35 (0.6) |
| WBC | 30 (0.5) |
| [Platelet](javascript:;) count | 28 (0.5) |
| Glucose | 29 (0.5) |
| Blood sodium | 27 (0.5) |
| Blood magnesium | 47 (0.8) |
| Blood [calcium](javascript:;) | 176 (3.1) |
| BUN | 30 (0.5) |
| Urine output | 194 (3.4) |
| LVEF | 625 (11.2) |
| Atrial fibrillation | 0 (0) |
| Myocardial infarction | 0 (0) |
| Coronary arteriosclerosis | 0 (0) |
| Hypertension | 0 (0) |
| Venous thrombus | 0 (0) |
| Anemia | 0 (0) |
| [Pneumonia](javascript:;) | 0 (0) |
| Diabetes | 0 (0) |
| Duodenal ulcer | 0 (0) |
| Gastric ulcer | 0 (0) |
| Gastrointestinal bleeding | 0 (0) |
| Gastritis | 0 (0) |
| Acute kidney failure | 0 (0) |
| Septic shock | 0 (0) |
| RAAS inhibitors | 0 (0) |
| Diuretics | 0 (0) |
| Inotropic agents | 0 (0) |
| Adrenaline receptor antagonists | 0 (0) |
| CCB | 0 (0) |
| PPIs | 0 (0) |
| Anticoagulants | 0 (0) |
| Antiplatelet drugs | 0 (0) |
| ICU mortality | 0 (0) |
| Hospital mortality | 0 (0) |
| 30-day mortality | 0 (0) |
| 90-day mortality | 0 (0) |
| 1-year mortality | 0 (0) |
| 5-year mortality | 0 (0) |
| 10-year mortality | 0 (0) |
| Hospital LOS | 0 (0) |
| ICU LOS | 0 (0) |
|  |  |
